# Relevant units of analysis for applied and basic research dealing with neglected transmissible diseases: The predominant clonal evolution model of pathogenic microorganisms

**DOI:** 10.1371/journal.pntd.0005293

**Published:** 2017-04-27

**Authors:** Michel Tibayrenc, Francisco J. Ayala

**Affiliations:** 1 Maladies Infectieuses et Vecteurs Ecologie, Génétique, Evolution et Contrôle MIVEGEC (IRD 224-CNRS 5290-UM1-UM2), Institut de Rercherche pour le Développement (IRD), Montpellier, France; 2 Department of Ecology and Evolutionary Biology, University of California, Irvine, California, United States of America; Beijing Institute of Microbiology and Epidemiology, CHINA

## Abstract

The predominant clonal evolution (PCE) model seeks to formulate a common population genetics framework for all micropathogens (namely, parasitic protozoa, fungi and yeasts, bacteria, and viruses). It relies on a definition of clonality that is only based on population structure features (namely, strongly restrained genetic recombination). Its clear-cut properties make it of strong interest for applied and basic research, since it permits the definition of stable, clearly delimited units of analysis below the species level: clonal genotypes and discrete genetic subdivisions (“near-clades”). These units of analysis can be used for clinical and epidemiological studies, vaccine and drug design, species description, and evolutionary studies on natural and experimental populations.

In this review, the evolutionary and population genetics background of the model will be only briefly mentioned, while considerable emphasis will be given to its practical significance for the study and control of neglected tropical diseases. The goal of the paper is to make this practical usefulness accessible to a broad audience of readers, including scientists who are not evolution specialists, such as epidemiologists, field scientists, and clinicians. For extensive developments about the evolutionary background of the model, see our previous papers [1–9]. Citations of these former articles lead to the many references quoted in them, which cannot be listed again here.

## Methods: Brief recall of the PCE model and how it has been designed

The PCE model of pathogenic microorganisms explores the population structure and evolution of whole, presently described species in their complete ecogeographical range in the long term. It aims at evaluating to what extent genetic recombination is inhibited in the species considered. It relies on two main approaches (namely, linkage disequilibrium [LD] analysis and phylogenetic analysis).

**Fig 1:**
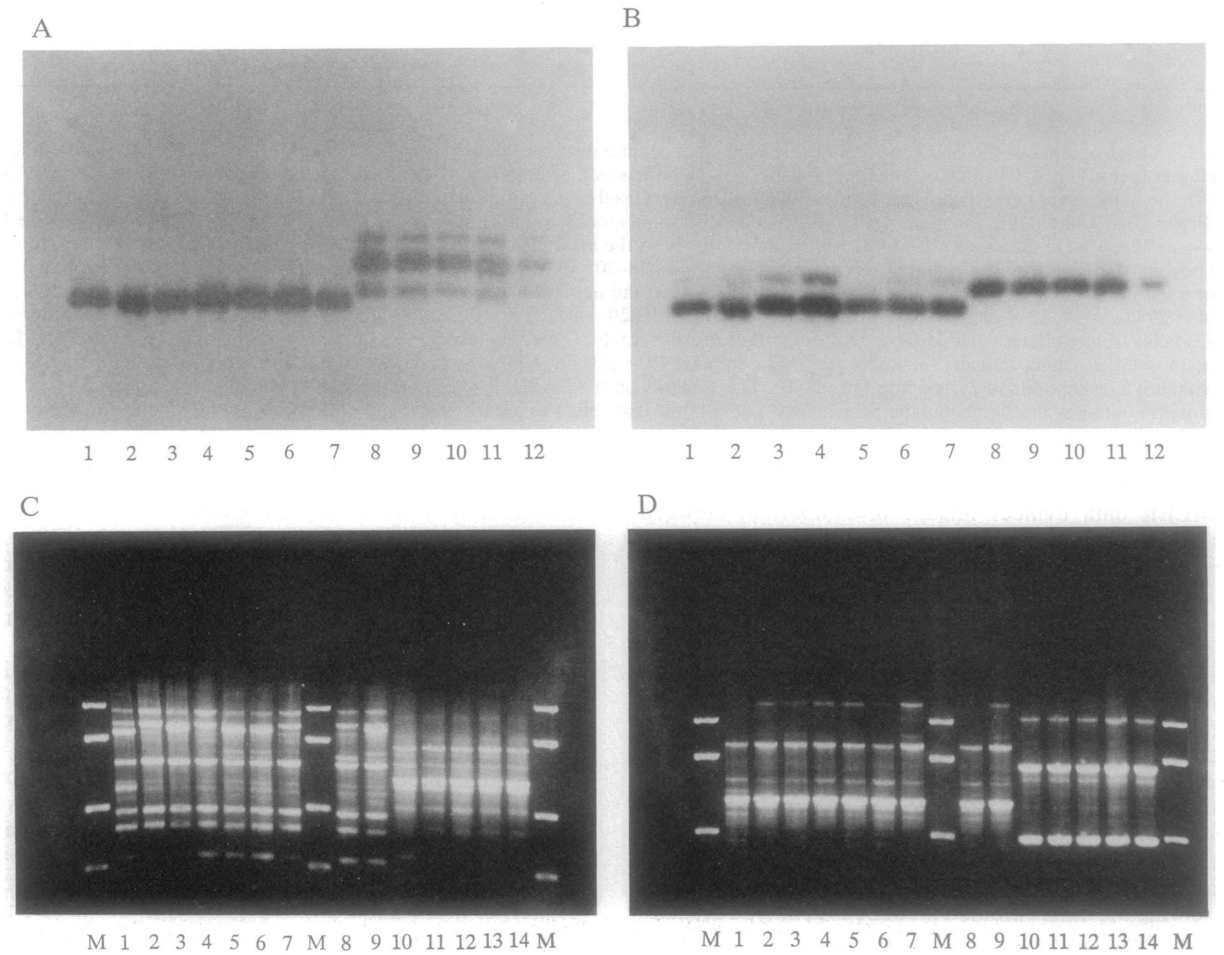
An extreme case of LD in *Trypanosoma cruzi*, the parasite responsible for Chagas disease. Top: two genetic loci (A and B) revealed by protein markers (multilocus enzyme electrophoresis [MLEE]); bottom: two genetic loci (C and D) revealed by DNA markers (Random Primed Amplified Polymorphic DNA). The four genetic loci are totally linked to each other: A 1–7 with B 1–7 with C 1–9 with D 1–9 on one hand, and A 8–12 with B 8–12 with C 10–14 with D 10–14 on the other. Cross genotypes (for example: A1 with D10, A2 with B8, or C3 with D13) have never been observed among more than 500 strains. The M lines in C and D are size markers (after [52]).

LD (see Fig 1 and S1 Glossary) is the very statistic designed to explore obstacles to genetic recombination in a given population. It is widely used by many authors specializing in pathogen population genetics [5, 9].

It is advised against using phylogenetic analysis with too strict cladistic demands in the case of microbial pathogens. As a matter of fact, even in species in which the PCE model is amply verified, occasional bouts of genetic recombination are most times recorded, Strict cladistic analysis is therefore improper, since the genetic isolation among clades sensu stricto is, by definition, complete. We have recommended a flexible phylogenetic analysis relying on a congruence principle: adding more relevant data reinforces the phylogenetic signal in the population under study (see Table 1 for congruence criteria).

**Table 1:** Congruence parameters. List of congruence parameters that support a growing phylogenetic signal and the presence of the “clonality threshold” in the species under study.

More genetic loci added
Genetic markers with more resolution added (for example: multilocus sequence typing [MLST], then whole genome sequencing [WGS])
Deep phylogenies revealed by large sets of multilocus markers and/or WGS
More individuals surveyed
More populations surveyed
Different populations at different places and times give similar population structure patterns
Parity between different kinds of genetic markers (for example, MLEE and random amplified polymorphic DNA [RAPD])
Parity between different phylogenetic approaches (for example: Unweighted Pair Group Method with Arithmetic Mean [UPGMA] and neighbor joining)
Parity between phylogenetic and nonphylogenetic approaches (for example: neighbor joining and STRUCTURE, which is a nonphylogenetic, unsupervised approach)

**Fig 2:**
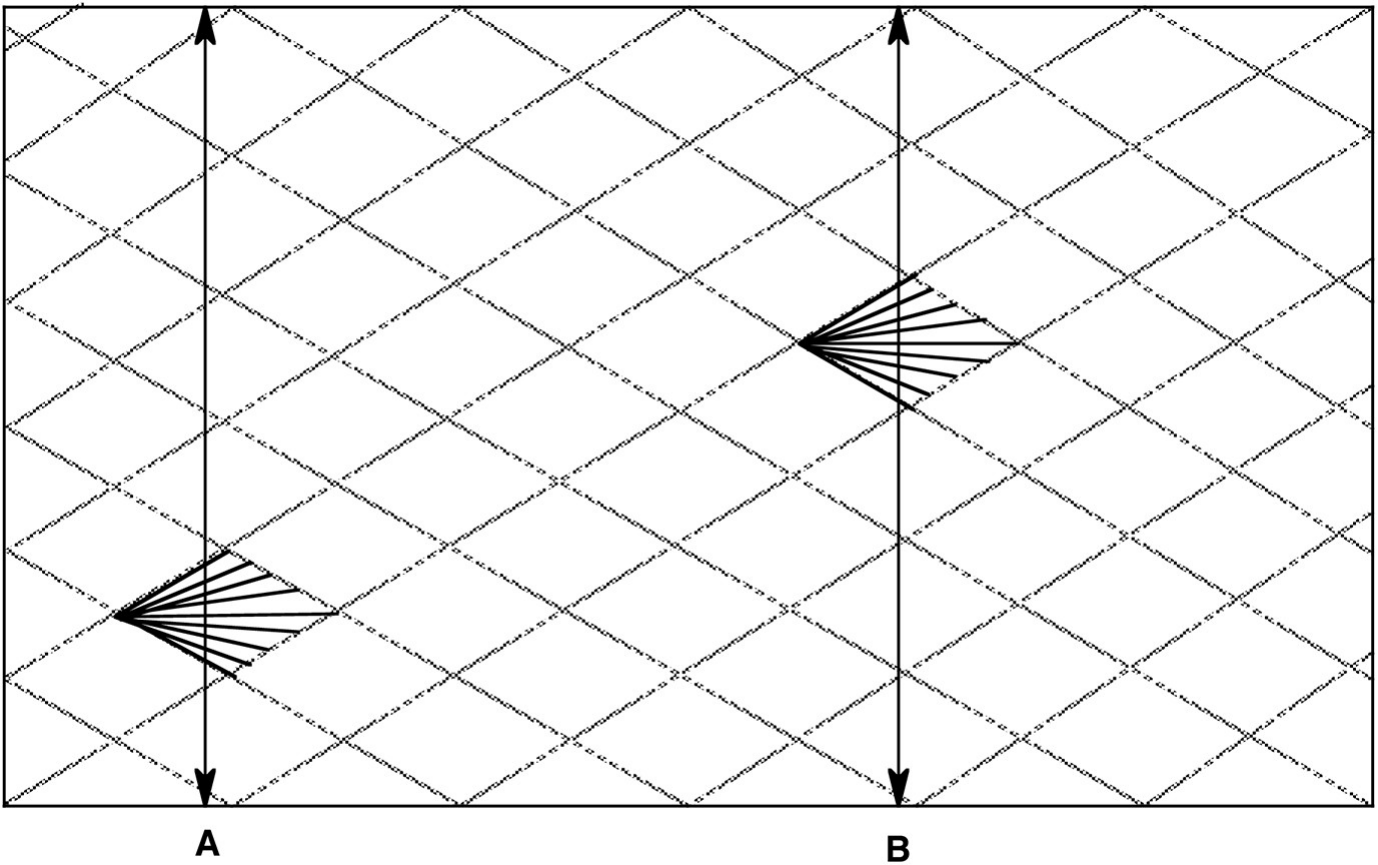
Evolutionary pattern of the semiclonal model [10]. In a predominantly recombining species, occasional bouts of clonality generate “epidemic” clones (symbolized by dark lines), the lifetime of which is limited to at most a few years; their genetic makeup then vanishes in the common gene pool. If samples are surveyed at times A or B, the presence of repeated clonal genotypes will increase the level of LD of the population, although this population is a predominantly recombining one. Growing phylogenetic signal and clonality threshold (see text above) are lacking in this situation (see ref. [16]).

This growing phylogenetic signal is the specific mark of the “clonality threshold.” We have coined this term to designate the point where PCE efficiently counters the effects of recombination. Beyond this point, the various genotypes observed within the species are bound to diverge irreversibly and lead to the individualization of the so-called “near-clades” (see below). Sometimes, deep phylogenies (the mark of genetic divergence in the long run) can be evidenced only by high-resolution methods, such as typing by large sets of genetic markers in the case of *Toxoplasma gondii* [7] or by WGS in *Neisseria meningitidis* [5]. The existence of such deep phylogenies is one of the most reliable manifestations of the clonality threshold. It is incompatible with the so-called “semiclonal” model [10]. This model corresponds to the pattern of occasional bouts of clonality in a recombining species (Fig 2). It states that the phylogenetic signal weakens and vanishes in the long term, due to the impact of genetic recombination.

The core strategy of our approach to looking for the clonality threshold has been to confront a huge amount of data dealing with parasitic protozoa (25 species), fungi (9 species), bacteria (32 species), and viruses (23 species and categories) through the in-depth analysis of more than 450 articles [9]. Most times, the analyzed papers had been designed by their authors with goals that were different from ours, and sometimes, we did not follow the conclusions of the authors. The most specific criterion in our approach has been that studies relying on different markers, different pathogen populations, and different ways of performing LD and phylogenetic analysis gave convergent results, according to the congruence principle described above. These congruent features were not apparent to the eyes of the authors of these articles for the reason that this field of research is highly compartmentalized and, again, because these various studies had different goals than ours. The final added value and theoretical strength of our study are the resultant of (and integrate) the individual fine added values of all the approaches developed in the many papers analyzed. This final added value does not rely on any new mathematical modeling, but rather on abundant, simple observations and extensive comparisons interpreted in the light of our PCE working hypothesis. As an example of our approach, splitstree [11] is a software aiming at designing a tree-like network structure when conflicting phylogenetic signals are apparent. STRUCTURE [12] is a nonphylogenetic, unsupervised approach in which no groupings are determined a priori, and the unit of analysis is the individual. Its use for pathogenic microbes has been criticized [13] since it is based on panmictic assumptions, which are virtually never observed in pathogens. Although splitstree and STRUCTURE rely on quite different approaches, and in spite of the restrictions expressed for the use of STRUCTURE in nonpanmictic organisms [13], both methods give convergent results with regards to the genetic structuring of many pathogen species. This strongly supports the robustness of these genetic structures, which stand after the analysis by two quite different methods.

The originality of our approach, therefore, lies in putting together a wealth of data that has never been considered jointly towards testing a unique working hypothesis (namely, PCE and its specific clonality threshold). Our analysis has remained as close as possible to the original data so that everybody can easily test our inferences by simply returning to these original data. This approach made possible the emergence of a convergent picture common to many different pathogen species.

**Fig 3:**
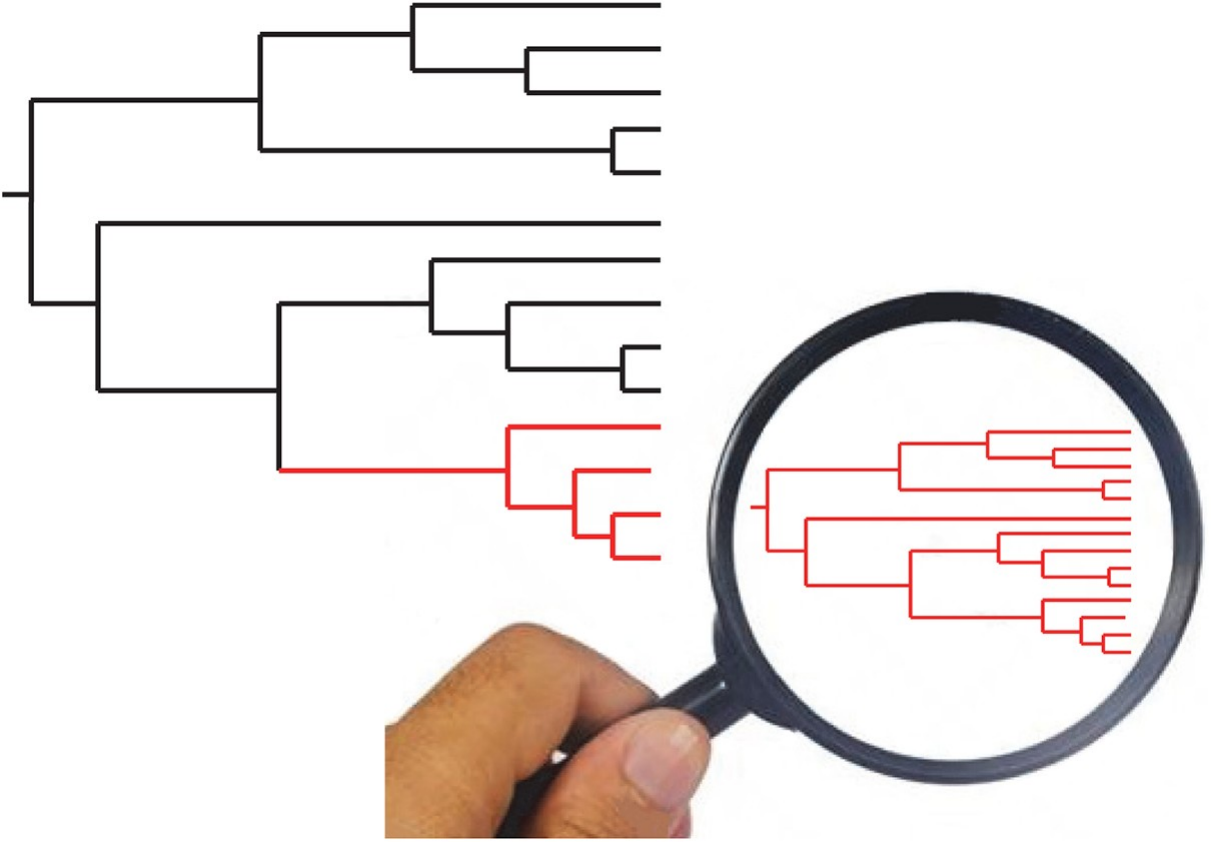
“Russian doll” model. When population genetic tests are performed with adapted markers of sufficient resolution within each of the near-clades that subdivide the species under study (large tree, left part of the figure), they reveal a miniature picture of the whole species, with the two main PCE features, namely, LD and lesser near-clades (small tree, right part of the figure). This supports the hypothesis that the near-clades are not potentially panmictic, biological species and rather that they also undergo predominant clonal evolution.

The main features of PCE at the level of a given species are a strong (statistically meaningful) LD; the widespread occurrence of clonal multilocus genotypes; the presence of discrete genetic subdivisions, which we have called “near-clades,” the reason for which is stated above (impossibility to use a classic cladistic approach for pathogenic microorganisms) as well as later (see paragraph below: “near-clades”) [5]; and a “Russian doll” pattern [6] (Fig 3) (namely, within each of the near-clades that subdivide the species under study, PCE still is verified, with LD, clonal multilocus genotypes, and lesser near-clades, up to microevolutionary levels in some instances). When exploring the within-near-clade population structure, one has to be aware that a lower evolutionary scale is considered. The resolution of the markers used should, therefore, be tuned up. If the marker has an insufficient resolution power, it could mimic genetic recombination, not because this feature is the case but because of a trivial statistic type II error (lack of power of the statistical test).

## Remarkable properties of the evolutionary entities revealed by the PCE model

LD is a direct consequence of restrained genetic recombination, and it is the specific statistical way to test it. It makes that the genotypes occurring at different loci are “stuck together” and are transmitted jointly, without being disrupted by genetic recombination (Fig 1). LD is the remarkable property that makes molecular epidemiology, strain typing, and epidemiological tracking possible. If LD is not present, MLGs—in other words, strains—are ephemeral, since they are frequently disrupted by genetic recombination. It is therefore pointless to try and follow them for epidemiological surveys. When LD is absent or weak, the only relevant units of analysis are individual genes.

Clonal MLGs and clonets are a direct consequence of LD. Clonal MLGs are very stable in space and time, constituting relevant targets for molecular epidemiology/strain typing. In many instances, they are sampled unchanged over broad geographical areas and long periods of time. However, clonal MLGs, excluding some exceptions, should not be considered as true clones (that is to say, perfectly homogeneous MLGs). This depends on the level of resolution of the marker used. For example, the MLG MON 1 of *Leishmania infantum* is monomorphic and behaves as a clonal MLG when MLEE is used. The application of microsatellites [14] and even more of a broad set of single nucleotide polymorphisms (SNPs) [15] reveals a considerable genetic heterogeneity within MON 1. Moreover, in [15], the “191 core group,” which is monomorphic when microsatellites are used, exhibits six discrete monophyletic lineages when SNPs are used. We have coined the term “clonet” for these clonal MLGs that appear as monomorphic for a given marker while more high-resolution markers reveal additional genetic polymorphisms within them [4]. The clonet concept is crucial when molecular epidemiology is concerned. If the level of resolution of the marker used is not high, the most recent common ancestor of the MLG to be characterized could be as ancient as several hundred years. This has to be taken into account in molecular epidemiology surveys. Such a marker is not appropriate at microeveolutionary levels (for example, surveying the spread of a pathogenic clone in a hospital at time intervals of a few weeks).

Indirect typing and specific markers: again, a direct consequence of LD; knowing the genotype at one genetic locus or a few loci makes it possible to predict the genotypes at all other loci of the MLG under study. In Fig 1, knowing the MLEE genotype A1 makes it possible to predict the MLEE genotypes A2 to A6 and B1 to B6 and the RAPD genotypes C1 to C9 and D1 to D9, if the overall genetic diversity of this population has been conveniently surveyed. The practical interest of this property is obvious: namely, the possibility to design specific markers (“tags”; [16]) for all clonal MLGs and near-clades. This would be impossible in the absence of LD, since the MLGs are ephemeral in frequently recombining species.

Near-clades: through phylogenetic analysis, stable, discrete genetic subdivisions are apparent in many pathogen species. However, both population genetics and phylogenetic analysis show that occasional bouts of genetic exchange and/or hybridization exist in almost all pathogen species surveyed until now. The occurrence of occasional recombination/hybridization is definitely included in the PCE model, which has always taken it into account [1, 3]. However, the term “clade” is inappropriate for such discrete subdivisions, since among strictly speaking clades, genetic isolation is absolute. We have forged the term “near-clade” for such genetic subdivisions that undergo occasional instances of genetic exchange [5]. The near-clades exhibit remarkable properties that make them appropriate units of analysis for applied and basic research. They are genetically discrete and very stable in space and time. They can be characterized by appropriate markers (“tags”; [16]) that are equivalent to synapomorphic characters in classical cladistic analysis.

A major obstacle for evaluating the impact of pathogens on the severity of infectious diseases is the immense genetic variability of pathogen species [17]. The near-clade concept makes it possible to address this question by using a limited number of relevant units of analysis. Instead of surveying an infinite number of pathogen genotypes, one will instead analyze a representative sample of genotypes of each near-clade that subdivide a given species.

Near-clades as a basis for describing additional new species below the species level: the fact that (in most pathogenic microorganisms) occasional genetic recombination occurs makes it impossible to describe below the species-level clades in the strict sense of the term. This renders the phylogenetic species concept [18] tentative or invalid. The near-clade model relaxes the cladistic demands. Near-clades evidenced below the species level can be taken as units to describe additional species, if the specialists of the species under study find it desirable and informative (see “Some illustrative cases”: the case of *Trypanosoma cruzi*).

Within near-clade genetic diversity: “Russian doll” pattern. In several major species analyzed by population genetics, such as *Tryp*. *cruzi*, the parasite responsible for Chagas disease, the presence of near-clades is not under much dispute. However, it has been hypothesized that, while gene flow between near-clades is “self-evidently” restricted (which amounts to saying that the results of any population genetic or phylogenetic study are self-evident), within near-clade genetic exchange is much more abundant than between near-clades [19]. We have proposed [6] the “Russian doll” model (see “Remarkable properties of the evolutionary entities revealed by the PCE model”) with the very goal of testing this hypothesis. The features revealed by the within near-clade Russian doll approach have the same properties as the features observed at the level of the whole species (namely, LD, stable MLGs, and lesser near-clades). However, one has to remember that the most recent common ancestor (MRCA) of each near-clade is more recent than the one of the whole species. In some extreme instances, the MRCA could be only a few hundred years old [15]. At such a microevolutionary level, extremely high-resolution markers should be used, and some properties of the PCE model may not be fully verified, because too limited genetic variability renders any test impossible.

The above summarized properties of the PCE model lead to a sharp conclusion: only in those species in which PCE has been adequately tested and in which the presumptions for it are sufficiently strong can molecular epidemiology and strain typing be reliably applied. As a matter of fact, if PCE features are not verified (which is the case, for example, in highly recombining bacteria such as *Helicobacter pylori* [20]), the classical units of analysis that permit molecular epidemiology and strain typing (clones and clonets, stable MLGs, and near-clades) have no stability in space and time and are therefore improper for any characterization attempt. This is why PCE constitutes the very theoretical basis of molecular epidemiology. This remarkable trait has not been pinpointed in a recent review on molecular epidemiology based on WGS [21]. Taking the PCE features for granted could be extremely misleading. It has to be emphasized that a majority of pathogenic microorganisms have not been conveniently analyzed with the PCE approach. For many species, the job still has to be done or, at least, completed (Table 2). In summary, the “clonality threshold” is also the “stable and discrete unit of analysis threshold,” beyond which relevant units of analysis (clones, clonets, and near-clades) can be characterized, labeled by indirect genetic typing, and followed up in the long run. Under this clonality/molecular epidemiology threshold, the relevant unit of analysis is neither the MLG nor the near-clade but rather the individual gene, since MLGs are ephemeral and soon vanish in the common gene pool.

**Table 2:** List of species. List of species explored for PCE features, according to the PCE definition exposed in the present article (see [9]).

Bacteria	Fungi	Parasitic protozoa	Viruses
*Bacillus anthracis* *	*Aspergillus fumigatus*	*Cryptosporidium andersoni* †	Adenovirus
*B*. *cereus* *	*Candida albicans* *	*C*. *hominis* †	Chikungunya §
*Bartonella bacilliformis* §	*C*. *dubliniensis* §	*C*. *muris* †	DENV *
*B*. *henselae* §	*C*. *glabrata*	*C*. *parvum* †	Ebola §
*B*. *quintana* §	*Cryptococcus gattii* *	*Giardia intestinalis* *	Echovirus-Enterovirus §
*Borrelia burgdorferi* §	*Cryp*. *neoformans* *	*L*. *braziliensis* *	HAV *
*Burckholderia pseudomallei* §	*Fusarium oxysporum* §	*L*. *infantum* complex *	HBV §
*Campylobacter coli*	*Penicillium marneffei*	*L*. *guyanensis* §	HCV *
*Enterococcus feacium*	*Pneumocystis jirovecii* §	*L*. *killicki* §	HEV *
*Escherichia coli* *		*L*. *lainsoni*	HIV-1 §
*H*. *pylori* *#*		*L*. *major* §	Influenza §
*Legionella pneumophila* §		*L*. *Mexicana* §	Maize streak virus
*Listeria monocytogenes* §		*L*. *peruviana* §	Measle virus §
*Mycobacterium bovis* *		*L*. *tropica* §	Picornavirus
*M*. *tuberculosis* *		*Plasmodium falciparum* *#*	Poxvirus
*N*. *gonorrhoeae*		*P*. *floridense* §	RABV §
*N*. *lactamica*		*P*. *vivax* *#*	ScoV (SARS),
*N*. meningitidis *		*T*. *gondii* *	SIV
*Pseudomonas aeruginosa* §		*Tryp*. *brucei*	SLCov
*P*. *syringae* §		*Tryp*. *brucei gambiense* *	VARV §
*Salmonella enterica* *		*Tryp*. *brucei rhodesiense*	VZV §
*S*. *typhi* *		*Tryp*. *congolense* §	WNV *
*Staphylococcus aureus* *		*Tryp*. *cruzi* *	
*Streptococcus mitis* §		*Tryp*. *evansi* §	
*Strep*. *oralis*		*Tryp*. *vivax* §	
*Strep*. *pneumonia* *			
*Strep*. *pseudopneumoniae* *			
*Strep*. *pyogenes* §			
*Vibrio cholera* §			
*V*. *parahaemolyticus* §			
*V*. *vulnificus* §			
*Xanthomonas campestris* §			

*: Species for which there is fair evidence for a PCE pattern.

§: Species for which there are clear indications for PCE, although additional research is needed.

#: Species for which PCE features definitely are not observed.

†: Species for which PCE features are not observed, although additional research is needed to confirm it.

For other species, additional research is definitely needed before hypothesizing their population structure.

## Some illustrative cases

We present hereafter a few illustrative cases taken from parasitic protozoa, fungi and yeasts, bacteria, and viruses. In each case, we explain to what extent the species under survey fits the PCE model and what the relevance of the case is for applied and basic research. The cases for many more species and the aspects of dealing with the evolutionary biology background of the model have been extensively presented in previous papers [1–9, 16].

### Parasitic protozoa

*Tryp*. *cruzi*: the agent of Chagas disease is a paradigmatic case of the PCE model. The whole species is monophyletic and is subdivided into six “discrete typing units” (DTUs) [16, 22, 23]. A seventh DTU, named Tc-Bat because it is specifically linked to bat hosts, has been recently described [24]. *Tryp*. *cruzi* DTUs perfectly fit the definition of near-clades [5, 6]. Indeed, they are discrete and very stable in space and time. Occasional hybridization [25] prevents one from considering them as real clades. However, this is fully compatible with the definition of the near-clades. *Tryp*. *cruzi* near-clades are widely encountered in close sympatry, including the same vector and the same mammal host [26]. Such a feature should provide ample opportunity for mating among the DTUs. However, *Tryp*. *cruzi* near-clades keep their genetic integrity in the long term and over vast geographical distances, as can be verified by retrospective studies going back up to the late 1970s and 1980s [27, 28] and the genetic characterization nowadays of ancient strain collections. Host, vector, and ecological and geographical distribution of *Tryp*. *cruzi* near-clades has been the theme of a recent, broad review [26]. The same authors [26, 29] have questioned the classification of *Tryp*. *cruzi* into six to seven near-clades. However, in 137 articles analyzed by them, which rely on a diversified panel of samples and genotyping methods, the authors have been able to successfully identify the near-clade type of 6,343 strains, which strongly supports the view that this near-clade classification is robust. PCE-specific properties of *Tryp*. *cruzi* near-clades (discreteness and stability in space and time) makes them choice targets for basic and applied research (for example, drug resistance evaluation) [30]. When molecular epidemiology is considered, the near-clades and clonal MLGs (clonets) can be characterized by indirect typing by a limited set of selected markers, which saves much time and effort [31]. A meeting of a panel of experts [23] discussed the possibility of describing as new species the six to seven near-clades (“DTUs”) that subdivide the species. As a matter of fact, these near-clades represent discrete, stable genetic entities with some phenotypic and epidemiological specificities [26]. It was decided that keeping *Tryp*. *cruzi* as a unique species was a better option [23]. However, the same debate still is pending about the *G*. *intestinalis* near-clades (“assemblages”), which are quite similar to the *Tryp*. *cruzi* near-clades from an evolutionary point of view and also present some phenotypic specificities [8]. Ultimately, the specialists concerned would decide whether it is relevant to describe new species or not.

Although genetic exchanges within *Tryp*. *cruzi* near-clades have been suspected by several authors [32–34], clear cases of Russian doll patterns are observed in *Tryp*. *cruzi* every time the samples are adequate [6, 9]. This is especially true for the one of the near-clades, designated as TCI, where additional subdivisions (lesser near-clades), namely TCI a-e, have been described [35] and have been corroborated by the use of various genetic markers [36]. Various other cases are listed in [9]. However, within near-clade population genetics and population structure still remains a nascent field of research and needs further exploration for *Tryp*. *cruzi* as well as for all other pathogenic microorganisms.

The case of *T*. *brucei*, the agent of Human African Trypanosomiasi, is less clear. The so-called “*T*. *brucei gambiense* group 1” is a clear case of PCE [5, 37]. When considering the other *T*. *brucei* groups, the balance between clonal propagation and recombination is still a matter of debate [2, 5]. Experimental meiotic recombination [38] is a classical feature of this species and most probably occurs in nature.

For the species *P*. *falciparum* and *P*. *vivax*, the results are definitely different from *Tryp*. *cruzi*. The agents of malaria do not meet the PCE criteria. This is most probably the result of obligatory mating and meiosis in the anopheline mosquito vector. This means that molecular epidemiology and strain typing would be misleading in the case of these species. However, as we proposed [2, 3, 7], clonal propagation, probably due to frequent selfing (that is to say the union of two genetically identical gametes), is observed in some populations of these parasites, which leads to the presence of unstable genetic subdivisions and population stratification [5, 7]. *Plasmodium* natural populations are neither homogeneous nor panmictic. This should definitely be taken into account when the distribution of genes of interest (pathogenicity and drug resistance) is analyzed.

### Fungi

*C*. *albicans* is subdivided into near-clades [5], which is corroborated by several genetic markers [39]. Phenotype diversity is linked to the near-clades [39]. Ubiquitous major “clades” (near-clades) are subdivided into various minor clades (Russian doll pattern) [39].

The “complex” *Cryp*. *gattii/neoformans* also shows a typical pattern of near-clades and Russian dolls, which is corroborated by various markers [5, 8].

### Bacteria

*E*. *coli* counts among the most demonstrative cases of PCE and near-clading [5]. The MLEE A, B1, B2, and D groups identified in the historical “ECOR” collection of strains by pioneer studies [40] have been fully corroborated, and their permanency as well, by many studies relying on various genetic markers [41]. Moreover, WGS and the use of 16,799 SNPs reveal a striking case of Russian doll pattern in the highly pathogenic lineage of *E*. *coli* ST131. This lineage is a very small subdivision of the species. It is additionally subdivided into three “clades” (lesser near-clades), each of them evidencing many additional subdivisions supported by high bootstrap values [42].

*M*. *tuberculosis* is considered to have a highly clonal population structure [43] and shows a clear near-clading pattern [9] with seven human “lineages” (near-clades) [44]. A Russian doll pattern is clear in the agent of tuberculosis. As a matter of fact, lineage 4, which is itself a tiny subdivision of the whole species, is subdivided into 10 “sublineages,” as evidenced by whole genome sequencing and the use of 9,455 SNPs. Some of these sublineages (lesser near-clades) are distributed worldwide [44].

*S*. *aureus* exhibits also typical PCE features (in particular, remarkable Russian doll patterns). Within the same “sequence type” (tiny genetic subdivision delimited by multilocus sequence typing [10]), additional, clear-cut “clades” (near-clades) can be evidenced by WGS [45–47].

WGS and the use of broad sets of SNPs appear to be master tools for researching such Russian doll patterns up to a microevolutionary scale. As an example, the most recent common ancestor of clade C in the *E*. *coli* pathogenic genotype ST131 is only 30–40 years old [42].

A PCE pattern is strongly supported in several other species surveyed by us (Table 2). However, for many, if not most, species, the job is far from completed. It should be completed before molecular epidemiology and description of relevant units of analysis are reliably applied to them.

In some cases, PCE features are so similar among radically dissimilar species that one can speak of “evolutionary twins.” This is so to the point that through a blind lecture, genetic data dealing with *Tryp*. *cruzi*, *G*. *intestinalis*, *C*. *neoformans*/*gattii*, and *E*. *coli* as examples could be confounded. The “evolutionary common denominator” of these four species is, therefore, very strong and leads to the same possibilities of identifying relevant units of analysis for applied and basic research.

### Viruses

Viruses definitely constitute a specific case. The smallness of their genome means that typing through WGS has since long become routine [21, 48]. Moreover, the evolution of their genome is much faster than that of bacteria, fungi, or parasitic protozoa, so shorter evolutionary scales are concerned. However, it has been hypothesized that PCE is a major feature of many viral species [5, 9, 49]. Several species exhibit typical PCE features, with the usual consequences for applied and basic studies. In viruses, like in other pathogenic microorganisms, the respective impact of clonal evolution and recombination is the key factor to be explored for evaluating the stability of relevant units of analysis in the long run. Two examples are discussed below (see also Table 2).

Dengue viruses (DENV) meet the PCE criteria. Their phylogenetic subtypes can be equated to near-clades [5, 9]. DENV near-clades exhibit Russian doll patterns and feature some phenotypic specificity, since they are statistically linked to serotypes [9].

HIV-1 is considered “highly recombining.” However, it exhibits stable groups (near-clades) M, N, O, and P, with phenotypic specificity and a Russian doll pattern within the M group [9]. However, due to many recombinant types [50], the stability of these groups in the long term has to be further explored by adequate studies.

## Conclusion

The PCE model appears to be the choice tool to explore the within-species genetic diversity of pathogenic microorganisms and its evolutionary, taxonomical, biological and epidemiological consequences.

New technologies such as megacomputing and high-throughput sequencing [21, 48] open promising avenues for molecular epidemiology in the broad sense: not only characterizing genotypes for epidemiological tracking (strain typing), but also identifying the relevant units of analysis, suitable for all studies addressing the below-species level: applied studies (vaccine and drug design, surveys dealing with clinical features and epidemiology, pathogenicity, resistance to drugs), as well as basic research: evolutionary studies of natural and experimental populations, description of new species below the level of presently described species.

Thanks to the major contributions of megacomputing, WGS, and massive use of SNPs, this field will progress considerably in the near future. The cost of sequencing has dropped dramatically. A bacterial genome can be fully sequenced in 2–5 days for no more than US$50 [47]. Routine WGS typing is now a reality for virus and bacteria [21, 48], which makes them “measurably evolving pathogens” [51], at time scales of a few tens of years only [42]. Such powerful technologies will be extremely valuable for confirming Russian doll patterns and hidden deep phylogenies, and hence, fully support PCE patterns in various species that need additional studies (Table 2).

According to the goal of the study, depending on the time and/or geographical scale to be considered, the PCE model makes it possible to tune up at will the level of resolution by considering either the first-level near-clades that subdivide the species considered or, subsequently, the lesser near-clades revealed by successive Russian doll subdivisions, up to the tiny microevolutionary levels evidenced by the use of thousands and tens of thousands of SNPs (a measurably evolving level on a scale of a few years or even less).

**Key learning points**

- Lack or rarity of recombination is the definition of genetic clonality and predominant clonal evolution (PCE).
- PCE leads to linkage disequilibrium, the propagation of stable multilocus genotypes, and the generation of genetic subdivisions that are stable in space and time.
- These genetic subdivisions should not be called “clades” but rather “near-clades,” because they are somewhat clouded by rare genetic exchange and therefore do not meet the strict definition of clade.
- Ubiquitous, stable multilocus genotypes (genetic clones) and near-clades constitute relevant units of analysis for basic and applied studies under the species level (strain typing, vaccine and drug design, evolution of natural and experimental populations, and species description).
- PCE features (see main text) are the specific manifestion of a “clonality threshold,” which is also a “molecular epidemiology threshold.” As a matter of fact, only beyond this threshold are the units of analysis stable enough in space and time to be reliable targets for exploring the consequences of intraspecific genetic variability of pathogenic microorganisms.

**Top five papers**

1. Feretzaki, F., J. & Heitman., J., 2013. Unisexual Reproduction Drives Evolution of Eukaryotic Microbial Pathogens. PLoS Pathog 9(10): e1003674.
2. Maynard Smith, J., Smith, N.H., O’Rourke, M., Spratt, B.G., 1993. How clonal are bacteria? Proc. Natl. Acad. Sci. USA 90, 4384–4388.
3. Perales, C., Moreno, E., Domingo, E., 2015. Clonality and intracellular polyploidy in virus evolution and pathogenesis. Proc. Natl. Acad. Sci. USA 112, 8887–8892.
4. Tibayrenc, M., Kjellberg, F., Ayala, F.J., 1990. A clonal theory of parasitic protozoa: the population structure of *Entamoeba*, *Giardia*, *Leishmania*, *Naegleria*, *Plasmodium*, *Trichomonas* and *Trypanosoma*, and its medical and taxonomical consequences. Proc. Nat. Acad. Sci. USA 87, 2414–2418.
5. Tibayrenc, M., Ayala, F.J., 2012. Reproductive clonality of pathogens: A perspective on pathogenic viruses, bacteria, fungi, and parasitic protozoa. Proc. Nat. Acad. Sci. USA 109 (48), E3305-E3313.

## Supporting information

S1 Glossary Glossary of specialized terms. (DOCX)
