# Supplementary material for: Relevant units of analysis for applied and basic research dealing with neglected transmissible diseases: The predominant clonal evolution model of pathogenic microorganisms
Source: PLoS Negl Trop Dis. 2017 Apr 27;11(4):e0005293. doi: 10.1371/journal.pntd.0005293 (PMC5407763; doi:10.1371/journal.pntd.0005293)
Supplement: S1 Glossary — (DOCX) [file pntd.0005293.s001.docx]

Glossary of specialized terms

Clade: Evolutionary lineage defined by cladistic analysis. A clade is monophyletic (it has only one ancestor) and is strictly isolated (it evolves independently) from other clades.

Cladistic analysis: A specific method of phylogenetic analysis that relies on the polarization of characters, which are separated into ancestral (plesiomorphic) and derived (apomorphic) characters. Only those apomorphic characters that are shared by all members of a given clade (“synapomorphic character”) are considered to be phylogenetically informative. For example, feathers are specific of the clade “birds” and are featured by all birds. They are synapomorphic characteristics of that clade.

Genetic recombination : Reassortment of genotypes occurring at different loci.

Linkage disequilibrium : nonrandom association of genotypes occuring at different genetic loci (figure 1). If there is no linkage disequilibrium in a given population (random genetic recombination), knowing the genotype of an individual at a given locus does not allow to predict the genotype at another locus. For example, in a human population within which mating occurs at random, for a given individual, knowing his/her ABO blood group says nothing about his/her Rhesus blood group. If the contrary is verified in a given population, it is an indication of linkage disequilirbium and suggests that genetic recombination has been hampered, with a strength that can be evaluated by various statistics.

Phylogenetic analysis : A branch of genetics that aims at reconstructing the evolutionary past and genetic relationships of taxa or of separate evolutionary lines.

Phylogeny: Evolutionary relationships between organisms or genes or molecules, often visualized as dendrograms of trees that are computed through various softwares.

Single nucleotide polymorphism: Polymorphisms or one-letter variations in the DNA sequence. SNPs contribute to differences among individuals, strains and populations. Widely used as high-resolution population markers.
